# Initial Study of the Onsite Measurement of Flow Sensors on Turbine Blades (SOTB)

**DOI:** 10.3390/mi15070877

**Published:** 2024-07-03

**Authors:** Lung-Jieh Yang, Chandrashekhar Tasupalli, Wei-Cheng Wang, Che-Yin Lee, Chi-Yuan Lee, Kunal G. Athikary, Jie-Xun Wu

**Affiliations:** 1 Department of Mechanical and Electromechanical Engineering, Tamkang University, New Taipei City 251301, Taiwan; chandrasekharamma25@gmail.com (C.T.); bmw001234567@gmail.com (W.-C.W.); 2 Department of Refrigeration, Air-Conditioning and Energy Engineering, National Chin-Yi University of Technology, Taichung 411, Taiwan; etonlee@ncut.edu.tw; 3 Department of Mechanical Engineering, Yuan Ze Fuel Cell Center, Yuan Ze University, Taoyuan 32003, Taiwan; cylee@saturn.yzu.edu.tw; 4 Department of Computer Science and Engineering, Presidency University, Bangalore Karnataka 560064, India; kunalathikary@gmail.com; 5 Department of Civil Engineering, Tamkang University, New Taipei City 251301, Taiwan; 157094@mail.tku.edu.tw

**Keywords:** sensor on turbine blade (SOTB), complementary metal-oxide semiconductor (CMOS), horizontal axis wind turbine (HAWT), onsite measurement

## Abstract

This paper presents a new framework using MEMS flow sensors on turbine blades (SOTB) to investigate unsteady flow features of a rotating wind turbine. Self-heating flow sensors were implemented by the U18 complementary metal-oxide semiconductor (CMOS) MEMS foundry provided by Taiwan Semiconductor Research Institute (TSRI). Flow sensor chips with a size of 1.5 mm × 1.5 mm were parylene-coated, output via a wireless data acquisition system (WDAQ), and mounted at the root, middle and tip of a 1.2 m diameter semi-rigid turbine blade of a 400 W horizontal axis wind turbine (HAWT). The instantaneous angles of attack (AOAs) of the SOTB were found to be 46~62°, much higher than the general stall AOA of 15°, but were accurate considering the normal detection of the flow sensors. The computational fluid dynamics (CFD) simulation of the HAWT was also compared with the SOTB output. The onsite measurement herein revealed that the 3D secondary flow increment, mostly obvious near the middle part of the turbine blades, degraded both the sensor and the turbine performance and initially justified the onsite measurement application.

## 1. Introduction

Due to the global trend for green energy and sustainability, wind turbine power generation (WTPG) has become one of the most popular technologies in the world. Whether an onshore or offshore wind turbine, taking the most common horizontal axis type as an example, the design of the turbine blades must be optimized as much as possible to ensure the high energy conversion efficiency of WTPG. Typical wind turbine blade design is based on the 5 MW wind turbine model provided by the National Renewable Energy Laboratory (NREL) to conduct aerodynamic experiments on scaled-down wind turbines in wind tunnels [1], or by using numerical analysis software FAST, also developed by NREL, for comparison. The results of the aforementioned wind tunnel test and FAST design simulation are also calculated and verified based on blade element momentum theory [2].

This article does not intend to revisit existing wind turbine blade design technology, but rather to introduce the on-site measurement technology of microelectromechanical systems (MEMS) [3,4,5,6] to WTPGs [7,8], as a new application concept for sensors on turbine blades (SOTB). SOTB is different from the conventional technology of using a traditional anemometer or Lidar to measure the wind speed at the location of the wind turbine to determine whether the wind turbine should start (cut-in) or feather (cut-out), etc. This new method of SOTB involves attaching flow sensors on the surface of the turbine blades and making them rotate together with the turbine blades. Therefore, the measured flow speed of the flow sensor is a combination of the freestream speed and the rotation speed of the turbine blade, further enhanced secondary flow increment. SOTB is believed to be a new method for measuring the three-dimensional (3D) dynamic flow velocity of rotating blades.

However, the flow sensor used in the SOTB of this work cannot be too bulky, otherwise the protruding part of the device installed on the blade will interfere with and change the local flow field of the turbine blade or even affect the efficiency of WTPG. The flow sensors available on the market, even if they claim to be MEMS products, are still not suitable for the SOTB purpose here. The flow sensor chip must be strictly packaged to protect it from external moisture, salt, and dust. Therefore, the size of the final packaged flow sensor is rarely less than 3.5 cm (packaged chip size of SB32 provided by Taiwan Semiconductor Research Institute (TSRI), Hsin-Chu). The size of the 3.5 cm flow sensor is insignificant compared to the 100 m long blades of MW-level giant wind turbines, but for the meter-scale blade of kW-level small wind turbines, it cannot be said to be very small.

Therefore, before exploring the application of SOTB technology, this article first introduces a MEMS flow sensor chip suitable for small wind turbine blades [9]. This MEMS flow sensor chip uses the UMC 0.18 µm 1P6M CMOS MEMS foundry service provided by TSRI (the minimum wire width on the chip is 0.18 µm). The working principle of the flow sensor is the so-called hot wire, which has been adopted by many prior applications [10,11,12]. Two polycrystalline thermal resistive detectors (RTDs), which are both fabricated on the silicon substrate of the chip and on the MEMS suspended thermal isolation area, are connected in a half-bridge configuration and biased by a DC voltage. When the airflow blows above the chip, a temperature difference is generated due to the different thermal insulation characteristics of the two RTDs on the above different places. The resistance value variations between the two RTDs are caused by the local temperature difference. Finally, the RTD resistance change is transferred to voltage change through the half bridge circuit according to its flow speed.

The size of the CMOS MEMS flow sensor in this article is only 1.5 mm × 1.5 mm. A self-made flexible print circuit board (PCB) can easily implement a single flow sensor with a reduced size of less than 1 cm. However, considering that electrostatic discharging (ESD) [13] may occur during self-mounting, wire bonding, and soldering processes during the above PCB self-making, the final production yield of the flow sensor is low. Standard dual-in-line-package (DIP) chip holders were adopted herein.

This article will start with the calibration test of the MEMS flow sensor placed stationarily in the wind tunnel, and then apply the SOTB concept to a 400W-class small wind turbine with a diameter of 1.2 m. Using wireless data acquisition (WDAQ) technology, the real-time flow speed of the SOTB can be collected. The signal will then compared with the computational fluid dynamics (CFD) results of the wind turbine as the conclusion of this initial study on SOTB.

## 2. Materials and Methods

### 2.1. The CMOS MEMS Flow Sensor Fabrication

**Figure 1:**
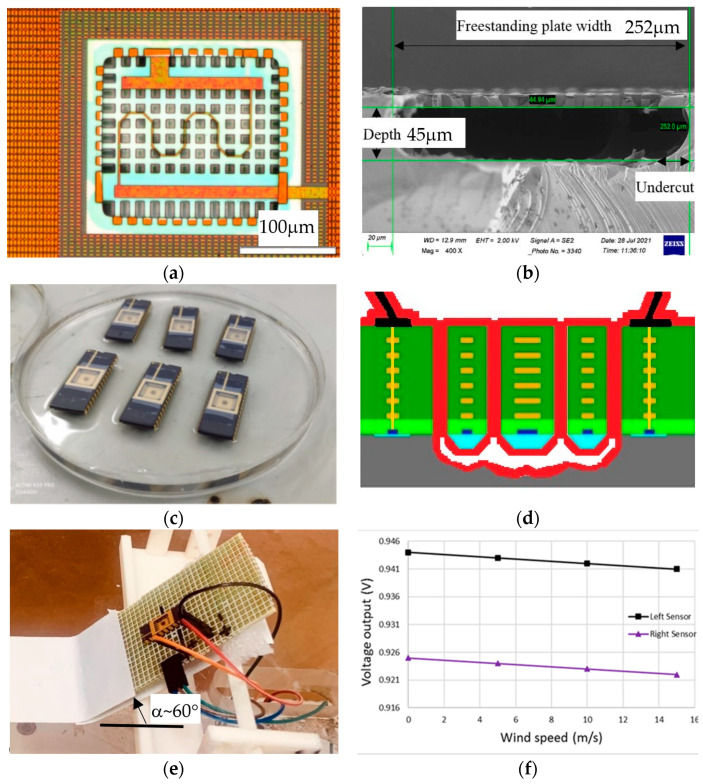
(**a**) Fabricated CMOS MEMS flow sensor die with orange-colored metal, light-brown-colored polysilicon and white-colored oxide; (**b**) SEM cross-sectional view of the sensor cavity [9]; (**c**) DIP-packaged sensor chips receiving parylene coating; (**d**) sensor schematic after parylene coating; (**e**) sensor tilting or with an inclined angle α in a wind tunnel; (**f**) output signals of the two half-bridged (upstream and downstream) RTDs with the same sensitivity of −138 µV/V/(m/s) [9].

The fabricated MEMS flow sensor die is shown in Figure 1a. After the CMOS MEMS foundry stage, the scanning electron microscopic (SEM) cross-section view is shown in Figure 1b. As Figure 1c shows, the flow sensor die is mounted and wire-bonded in the grooved well of the DIP. Figure 1d additionally shows the cross-section schematic of the sensor chip after being coated with parylene to prolong the lifetime [14]. Herein, the whole of the CMOS MEMS chips were coated with 10 μm parylene, which has a proven ability to insulate against moisture and dust, even against the ESD problem, although the sensitivity value is reduced from −138 µV/V/(m/s) to −36.6 µV/V/(m/s). Parylene coating is also a foundry service provided by Larchi Company (New Taipei City, Taiwan).

The completed CMOS MEMS flow sensors were calibrated by a low-speed wind tunnel with a wind speed of 0~15 m/s. The wind tunnel test section is 30 cm × 30 cm × 100 cm. The flow sensor was mounted on a 60°-inclined board in Figure 1e. With an application of a bias voltage of 1.8 V for the half-bridge RTDs (1 kΩ for each), the overall self-heating power is 1.62 mW (milliwatt) from the RTDs themselves. Figure 1f presents the output voltages vs. wind speed of the upstream (left) and downstream (right) half-bridged RTDs. Both flow sensors have the same sensitivity of −138 μV/V/(m/s) without parylene coating. The authors also tested the orientation effect of the flow sensor by changing the inclined angle α within 30~60°. This sensor orientation effect will degrade the output sensitivity by about 20~40%, and its influence on the onsite measurement will be discussed at the end of this paper.

### 2.2. A Commercialized Wind Turbine and Its Testing Run

To conduct the onsite measurement, a horizontal axis wind turbine (HAWT) was used (DB-400 model, Digi-Sine Company, New Taipei City, Taiwan). This wind turbine claimed to generate a rated power of 400 W. The technical specifications of the DB-400 turbine are as mentioned below:

- The blade diameter of this wind turbine is 1.2 m.
- The total weight of the blade and hub is 10 kg.
- It can start rotating from around 2 to 2.5 m/s and generate electrical power.
- It consists of a permanent-magnet brushless-motor for power generation with high voltage protection; for a battery of 14.8 V, the overcharge current protection is 20 A.
- The blade is made of a nylon and glass fiber composite.
- The turbine is attached with a maximum power point tracking (MPPT) braking system to prevent damage from natural disasters.

A large wind tunnel test was conducted in Tamkang University Wind Energy Research Center (TKU-WERC). The turbine started rotating at 2.5 m/s (cut-in speed). Following that, the wind speeds were incremented by 1 m/s to ensure uniformity in the datasets. The wind tunnel testing was carried out for wind speeds ranging from around 2.5 to 15 m/s. The RPM of the turbine rotation vs. wind speed was measured by a high-speed camera or frequency counter. The power generation for charging a 12 V battery as a load was also recorded. The maximum output power was measured as 50 W, only 12.5% of the rated power of 400 W.

**Figure 2:**
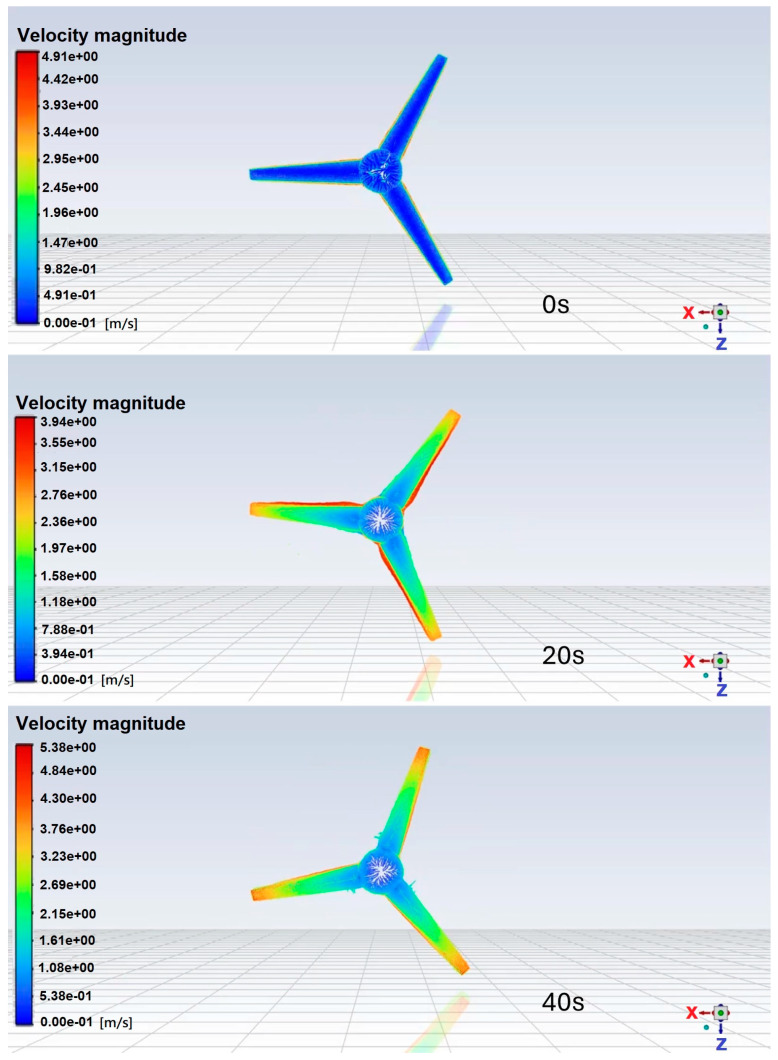
CFD simulation of an HAWT subject to 2.5 m/s freestream wind speed at 0, 20, and 40 s.

To more clearly describe the operation of the HAWT or HAWPG, a CFD simulation for demonstrating the turbine rotation subject to the wind blowing is shown in Figure 2. ANSYS Fluent 2022 R1 software was used for simulation. The related settings of the numerical simulation are as below.

(1) The wind turbine model is generated by SolidWorks software.
(2) The distance from the inlet to the turbine head is 3d, and distance from the outlet to the turbine tail is 5d, where d is the blade diameter (1.2 m) for the CFD computation domain.
(3) The transient k-ε turbulence model was employed, which belongs to the Reynolds average Navier–Stokes simulation (RANS) model. This model is suitable for simulating the mean flow characteristics in turbulent flow conditions and allows the authors to conclude the turbine blade situation in relation to the kinetic energy and turbulent dissipation rate.
(4) The inlet wind speed was set as 2 and 2.5 m/s according to the specification of the wind tunnel.
(5) The outlet pressure was set as ambient pressure.

Figure 2 shows the front views of the simulated velocity distribution of the HAWT subject to a freestream wind speed of 2.5 m/s at 0, 20, and 40 s. These CFD flow fields will be explained in relation to the CMOS MEMS flow sensor outputs later on.

**Figure 3:**
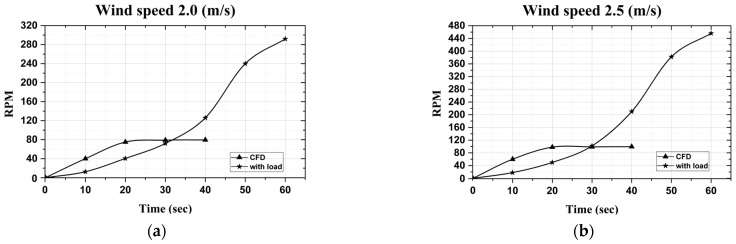
The comparison of RPM between wind tunnel testing with load and CFD according to (**a**) wind speed 2.0 (m/s) and (**b**) wind speed 2.5 (m/s).

Turbine testing at different wind speeds is shown in the curves in Figure 3. It reveals that the RPM increases with an enhancement of the wind speed. Comparing with the CFD, it can be successfully matched for the results, showing the relationship between RPM and the wind speed. This curve supports the theory that computational fluid dynamics (CFD) in ideal conditions are appropriate for turbine testing and provides a method to infer values from the sensor data.

As the result of the rotating testing under load, the RPM reaches 126 at a wind speed of 2.0 m/s and 221 at a wind speed of 2.5 m/s at the 40th second. Our observations indicate that the turbine’s RPM under load gradually stabilizes over time. In contrast, the CFD simulation shows a rapid increase in RPM due to the absence of load or external environmental factors. At flow speeds of 2.0 and 2.5 m/s, Figure 3 demonstrates that the RPM values for both the CFD simulation and the experimental wind tunnel testing converge at approximately 30 s. This point serves as a reference for comparing CFD and onsite measurements under similar conditions.

### 2.3. Wireless Data Acquisition System (WDAQ)

A wireless data acquisition system (WDAQ) was necessary for the signal wire connection of the onsite CMOS MEMS sensors on the rotating turbine blades. There are few wireless data acquisition methods available for collecting data or analyzing the collected data from aircraft system states [15]. The device used is portable and can be carried into an aircraft with the help of some crew members or the pilot. Another data acquisition system is applied in clinical settings, where this device is used for studying sleep, monitoring for brain emergency and in other clinical studies [16]. These prior systems are too bulky, and prompted the authors to develop their own. The WDAQ architecture was designed based on real-time constraints, and the full device was developed using appropriate electronic components, such as the Arduino-Nano microcontroller and the ESP8266 wireless communication module.

**Figure 4:**
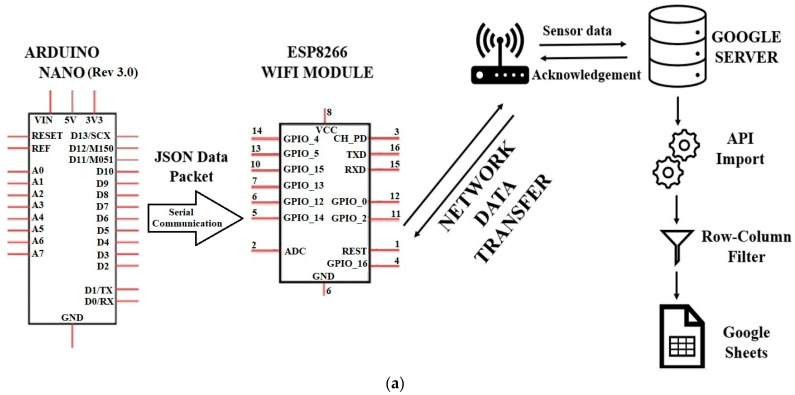

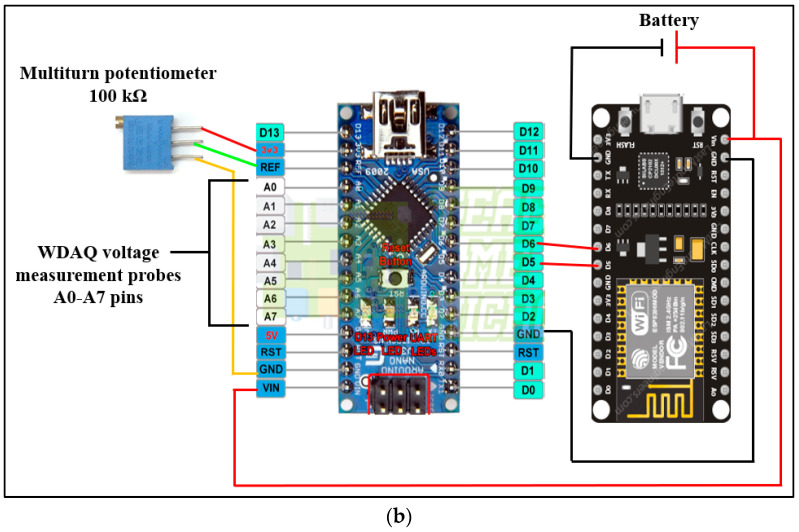
(**a**) Working process of WDAQ; (**b**) WDAQ circuit layout.

The ESP8266 is a simple and less expensive Wi-Fi module with microcontroller capability. It can easily communicate with other microcontrollers, such as Arduino (8 analog channels herein) on a 2.4 GHz Wi-Fi network. It has the advantage of connecting to Wi-Fi networks and being able to communicate with other devices remotely with ease [17]. By implementing the ESP8266 WiFi module, the collected data are sent as Java small object notation (JSON) from one application to another and all the data are stored structurally to avoid sending garbage data randomly to the computer. JSON is a simple format for transmitting data and helps to send the readable data in small packets [18]. The ESP8266 is connected to a network through which it can communicate with Google Sheets using the application program interface (API). After successful communication, a code is deployed on the Google App script to read the data from ESP8266, and the data are entered into the exact rows and columns in real time. The working process of the WDAQ is shown in Figure 4a, and a multiturn potentiometer in Figure 4b was used to adjust the reference voltage.

The fabricated WDAQ was calibrated and compared with the commercial data acquisition system DAQ 970A. The efficiency comparison is presented in Table 1. In this comparison, the WDAQ was connected to a power supply, and random values were generated and sent to both the WDAQ and DAQ 970A. Three trials were conducted to verify the efficiency of the WDAQ to match that of the DAQ 970A. Their repeatability was observed. The WDAQ demonstrated an exceptional efficiency of approximately 96.9% to 99.75% compared to the commercial DAQ. The slight errors observed are likely due to the slight time delay during the start of data recording.

The efficiency of the WDAQ relative to the commercial DAQ970A has been thoroughly evaluated. In terms of cost, the WDAQ offers a significant financial advantage. The estimated cost for WDAQ is approximately USD 50, compared to about USD 10,000 for the DAQ970A. This substantial cost difference, combined with the WDAQ’s comparable performance, underscores its potential as a cost-effective alternative for data acquisition in various applications. Thus, the WDAQ not only reduces investment costs but also provides efficient data collection capabilities, making it a viable choice for budget-sensitive projects.

This self-made WDAQ effectively provides 6~8 data points per minute. Each data point has 7 decimal digits, and the voltage is adjusted in advance. The signals are averaged and then wirelessly transmitted within a range of 20 m, which can be applied to the wireless transmission of measurement signals from the flow SOTB. Although the wind turbine blades rotate during operation, it is generally regarded as an unsteady flow field problem. However, under the periodic assumption that the rotation RPM is constant, it can be regarded as a quasi-steady flow field. The output voltage of the sensors on blades and the WDAQ on the turbine center cone are fast and applicable enough to respond to the changing flow field.

### 2.4. SOTB Test

**Figure 5:**
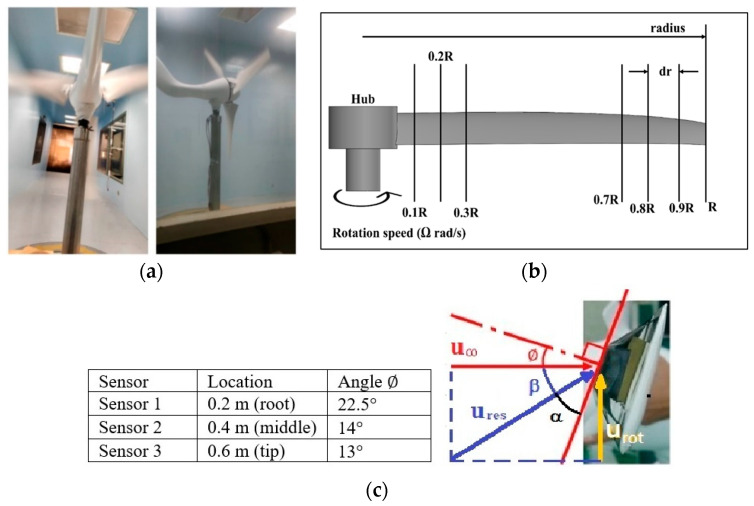
Nomenclature of 2D subdivision of turbine blades: (**a**) turbine rotating in the wind tunnel; (**b**) along the blade length; and (**c**) about the tilt angle ∅, the resultant angle β and the instantaneous AOA α.

The nomenclature of the two-dimensional (2D) subdivision of turbine blades is shown in Figure 5. The relationships between the tilt angle ∅, the resultant angle β and the instantaneous AOA α are shown in Figure 5c and analyzed by the following equations in terms of freestream wind speed $u_{\infty }$, resultant velocity $u_{res}$, and rotational speed $u_{rot}$.

(1) $$\beta ={cos}^{-1}\left(\frac{u_{\infty }}{u_{res}}\right)$$

(2) $$u_{res}=\sqrt{u_{\infty }^{2}+u_{rot}^{2}}$$

(3) α=90°−∅−β

**Figure 6:**
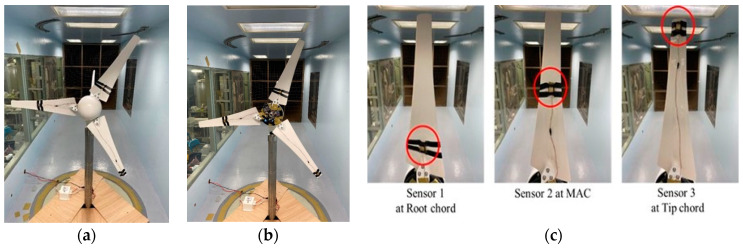
(**a**) Full test setup of wind turbine with CMOS MEMS sensors marked with red circles; (**b**) WDAQ inside the central cone; (**c**) CMOS MEMS sensors placed at different locations. According to the nomenclature of Figure 5, Sensor 1: 0.33 R, ∅ = 22.5°; Sensor 2: 0.67 R, ∅=14°; and Sensor 3: 1.0 R, ∅=13°. R = 0.6 m.

The full assembly of turbine blades, along with CMOS MEMS sensors and WDAQ inside the large wind tunnel, is shown in Figure 6. The wind tunnel test section is 200 cm × 200 cm × 600 cm. The CMOS MEMS sensors are positioned in three different locations on the blade surface: Sensor 1 is near the blade root, Sensor 2 is at the middle or the mean aerodynamic chord (m.a.c.) and Sensor 3 is near the tip. All three sensors are placed on the pressure side, where the flow may be less dependent on the AOA and wind speed. Consequently, the sensors detect confined flow features and provide limited flow information. However, if the sensors are positioned on the suction side, the much lower-speed separation eddies could negatively affect the sensor output.

The sensors were placed at these locations to investigate the local velocities of the blade at different wind speeds. As previously mentioned, the sensors were positioned in an array on each blade to precisely predict the blade performance, velocity characteristics and the blade health. For conducting the on-site measurement on this wind turbine experiment, the wind tunnel facility from TKU-WERC was used.

## 3. Results and Discussion

### 3.1. SOTB Data

**Figure 7:**
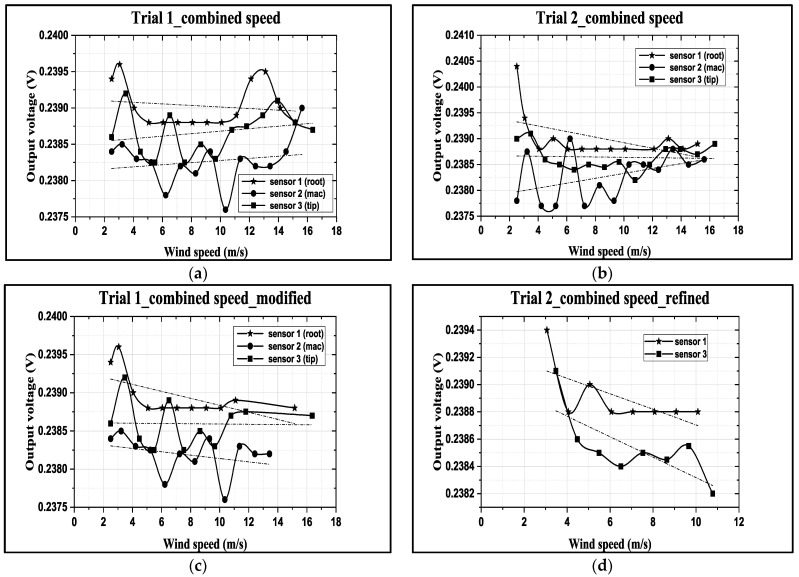
(**a**,**b**) Two trials of output voltage of 2 CMOS MEMS flow SOTB vs. actual combined speed in the wind tunnel; (**c**) Trial 1, refined output voltage of 3 CMOS MEMS flow SOTB vs. actual combined speed in the wind tunnel; (**d**) Trial 2, refined output voltage of SOTB vs. actual combined speed in the wind tunnel within a narrower speed region between 3 and 10 m/s.

From the WDAQ, the output voltage data were collected from three CMOS MEMSsensors. The received signals were DC voltage values. While incrementing the wind speed in the wind tunnel, a one-min interval was given to allow the wind turbine to stabilize and exhibit the proper rotating performance. To ensure the reliability of the output data from the CMOS MEMS sensors, two trials (Trial 1 and Trial 2) were conducted for the same operating conditions, as shown in Figure 7.

The nonlinear outputs of the flow sensors were locally linearized to determine their respective sensitivities. According to the working principle of thermal flow sensors [9], the sensitivities of these curves in Figure 7a,b should be negative. However, this is not true for the outputs of Sensor 2(●) or Sensor 3(■), which fluctuated most significantly among the three sensors and had positive sensitivity. Proper refinement of the data was conducted.

Firstly, observe Trial 1 of Figure 7a. The authors dropped the overshoot parts of Sensors 1(★) and 3(■) at the higher speed region and refined the data, as shown in Figure 7c. Sensor 3(■) at the blade tip experienced higher speeds and had lower sensitivity. However, Sensor 3’s sensitivity (−1.76 μV/1.8 V/(m/s)) was small for an air speed of up to 16.4 m/s, compared to Sensor 2(●) at the middle position, which had a sensitivity of −22 μV/1.8 V/(m/s) for an air speed of up to 15.6 m/s. Additionally, Sensor 1 had a sensitivity of −45.9 μV/1.8 V/(m/s) at the root for a drifting air speed from 15 to 15.2 m/s, as shown in Figure 7c.

The outputs of Sensor 2(●) in Trials 1 and 2 of Figure 7a,b fluctuated significantly with air speed and were left for further discussion. The authors ignored the data of Sensor 2(●) in Figure 7b.

We can continue to examine Trial 2 and discuss the outputs of Sensors 1 and 3 in the 3~10 m/s region in Figure 7d. Comparing the output of Sensor 1(★) at the root with the output of Sensor 3(■) at tip, both outputs have similar sensitivities (−56.8 and −75 μV/1.8 V/(m/s)) with 0.0002 V offset difference. Hence, the authors averaged the outputs of Sensors 1 and 3 to obtain a common sensitivity of −65.9 μV/1.8 V/(m/s) under a 1.8 V DC bias. Ideally, all CMOS MEMS sensors should have the same sensitivity, However, only Sensors 1(★) and 3(■) had similar output behavior. The average sensitivity equation derived from the calibration of Sensors 1(★) and 3(■) in more uniform flow fields on the same turbine blade is shown below.

V_out_ = −65.9 [μV/1.8 V/(m/s)] ×1.8 V ×u_res_ [m/s] + 0.2392 [Volt] (4)

In the following, a more detailed discussion about the instantaneous angle of attacks (AOAs) for the three CMOS MEMS sensor locations will be provided, and will reveal that Sensor 2 is a stranger case than other two sensors.

### 3.2. Consideration of Instantaneous AOAs of the Flow Sensors

Referring to Equations (1)–(3), the authors calculated and summarized the instantaneous AOA α as 46~62° in Table 2 and Table 3 for Sensors 1–3, subject to different freestream wind speeds (3~10 m/s). Herein, the instantaneous AOA α at all sensor locations on the turbine blade were much larger than the general stall angles 10°~15° in the aerodynamic literature. Therefore, flow separation happened in all three cases of Sensors 1~3. Referring to ref. [19] regarding the high AOAs, the lift coefficients did not really drop to zero, but approached about 1.1 at AOA = 45° for the NACA 0012 airfoil. Recently, a the similar trend has also been shown for the NACA 0021 airfoil at AOA = 40° [20].

After re-checking the flow sensor signals in Figure 7, it can be seen that Sensor 3 (■) obviously behaves normally and better than Sensors 1(★) and 2(●). In contrast to the rotational speed in Table 2 and the instantaneous AOA values calculated in Table 3, if the authors regard the higher AOAs as indicating the stall condition of flow fields for the wind turbine, Sensor 2 had most values of AOA > 60°. Sensor 1 had only two data points with AOA > 60°, and Sensor 3 had none. Hence, the stall comparison of flow field appropriateness shows that Sensor 3 > Sensor 1 > Sensor 2, suggesting that the turbine geometry configuration at the location of Sensors 1 and 2 may need modification if all the flow sensors are functioning correctly. In other words, it demonstrates that the CMOS MEMS flow sensors developed in this paper effectively evaluate the flow field appropriateness of small wind turbine designs.

### 3.3. Consideration of the 3D Secondary Flow Increment of the SOTB

To ensure that the sensor works well, a CFD flow simulation clarifies the turbine performance and the local flow fields around the CMOS MEMS flow SOTB. The greatest difference between the 2D flow velocity in Figure 5c and the 3D flow velocity is the spanwise increment ${\vec{u}}_{span}$ and the streamwise increment ${\vec{u}}_{stream}$, induced by the 3D effect. Both of these velocity increments are shown in Table 4. Generally, the flow field is mostly 3D at the root and tip of rotating blades, although the flow field in the middle of the rotating blade also exhibits 3D features, including wind speed, rotating blade speed, and spanwise speed. These 3D velocity increments will influence the detectability of the flow SOTB in this work.

Regarding Sensor 1 at the blade root, only the freestream speed ${\vec{u}}_{\infty }$ is important. The rotation speed ${\vec{u}}_{rot.}$ is not apparent, and neither the spanwise increment ${\vec{u}}_{span}$ nor the streamwise increment ${\vec{u}}_{stream}$ is significant.

Regarding Sensor 2 for the blade middle position, the most complicated case, not only are the rotation speed ${\vec{u}}_{rot.}$ and the freestream speed ${\vec{u}}_{\infty }$ important, but the spanwise increment ${\vec{u}}_{span}$ and the streamwise increment ${\vec{u}}_{stream}$ also cannot be ignored. As the CFD local flow fields show, the apparent thickness of the turbine blade generated the streamwise increment ${\vec{u}}_{stream}$ and the 3D tip vortex generated the streamwise increment ${\vec{u}}_{stream}.$ After impinging on the turbine blade for a certain moment, the flow is separated into upward or downward branches. The upward branch causes a positive increment ${\vec{u}}_{stream}\;\mathrm{to}\;\mathrm{the}\;\mathrm{freestream}\;\mathrm{speed}\;{\vec{u}}_{\infty },$ and thereby decrease the instantaneous AOA with respect to Sensor 2. On the other hand, the downward branch causes a negative increment ${\vec{u}}_{stream}\;to\;the\;freestream\;speed\;{\vec{u}}_{\infty },$ and it therefore increase the instantaneous AOA with respect to Sensor 2. Both the upward and the downward branches will alter the output sensitivity of Sensor 2 due to the sensor orientation effect mentioned in Section 2.1. In particular, for the former upward branch, if the decreased AOA is less than 30°, there may be no sensor voltage output at all. The sensor orientation effect may explain why the onsite measurement flow sensors in Figure 7 are not ideal.

Regarding Sensor 3 at the blade tip, the 3D tip vortex is obvious due to the finite span of the wind turbine blade. Even though the tip vortex flow is more complicated than the freestream uniform flow ${\vec{u}}_{\infty }$, the apparent velocity increment is ${\vec{u}}_{span}.$ It confines and oscillates within a narrow range inside the tip vortex region, and it will not strongly change the instantaneous AOA about the sensor and its output sensitivity. Hence, the authors can have an acceptable onsite flow sensor measurement in Figure 7 similar to the sensor output characteristics calibrated in the wind tunnel.

From the above CFD result discussion in Table 4, it is necessary to pay attention to the inclined angle or the instantaneous AOAs of the turbine blade design, especially around the middle position. The authors suspected that the inclined AOA of the SOTB might sometimes alter to be less than 30°, causing the sensitivity to degrade by 3D secondary flow increments including ${\vec{u}}_{span}$ and ${\vec{u}}_{stream}$, which were produced by the improper design of the turbine blade at the middle position. Restated, the flow SOTB signal fluctuation herein reveals the inappropriateness of turbine blade design at the middle position. From the authors’ point of view, the tilt angle ∅ at the middle position in the nomenclature of Figure 5 should be adjusted between the current design value of 14° and the root location 22.5°. The blade design modification only reduces the corresponding instantaneous AOA to somewhat smaller than 60°, but restrains the flow separation of the turbine flow field and ensures the normal sensing of the SOTB.

In summary, the application scenario for using the flow SOTB is to observe whether all the flow sensor outputs have similar trends and sensitivities. If the output of any one of the sensors is abnormal, the blade geometry at the sensor location may need to be improved. The HAWT in this study, with only 12.5% of power takeoff efficiency, aims to enhance turbine geometry with the help of the flow SOTB technique in the future.

## 4. Conclusions

(1) According to the new concept of SOTB in this article, three self-developed CMOS MEMS flow sensors were successfully mounted and provided flow speed signals via WDAQ. The sensor output in the middle of the turbine blade exhibited significant abnormalities.
(2) The instantaneous AOAs of the three flow sensors were found to be within 4~62°. These high AOA values indicate the complexity of the turbine flow field but are beneficial for the normal operation of flow sensors.
(3) A CFD simulation was conducted regarding the same HAWT model, revealing that the 3D secondary flow increment at the middle position of the turbine blade is significant. Due to the improper blade design, the upward branch may degrade both the signal output of SOTB and the HAWT performance.
(4) The application scenario using the flow SOTB is used to observe whether all the flow sensor outputs have similar trends and sensitivities. If the output of any one of the sensors is abnormal, the blade geometry at the sensor location may need to be improved.

## Figures and Tables

**Table 1:** Efficiency of WDAQ with respect to commercial DAQ970A.

Trial	WDAQ	DAQ970A	Efficiency
1	0.4376409	0.4387333	99.75%
2	0.4294499	0.4399238	97.62%
3	0.4254039	0.4386157	96.99%

**Table 2:** The rotational speed of the three flow sensors with respect to different u_∞_ and RPM.

u_∞_ (m/s)	3	4	5	6	7	8	9	10	11	12	13	14	15
RPM	28	32	36	40	44	52	56	64	68	76	80	92	104
ω_rot_ (rad/s)	2.932	3.351	3.770	4.189	4.608	5.445	5.864	6.702	7.121	7.959	8.378	9.634	10.891
u_rot_ (0.2 m)(m/s)	0.586	0.670	0.754	0.838	0.922	1.089	1.173	1.340	1.424	1.592	1.676	1.827	2.178
u_rot_ (0.4 m)(m/s)	1.173	1.340	1.508	1.676	1.843	2.178	2.346	2.681	2.848	3.184	3.351	3.854	4.356
u_rot_ (0.6 m)(m/s)	1.759	2.011	2.262	2.513	2.765	3.267	3.518	4.021	4.273	4.775	5.027	5.780	6.535

**Table 3:** Instantaneous AOA information about the three sensors on the rotating turbine.

Sensor 1 (r = 0.2 m, ϕ = 22.5°)				Sensor 2 (r = 0.4 m, ϕ = 14°)				Sensor 3 (r = 0.6 m, ϕ = 13°)			
u_∞_ (m/s)	u_res_ (m/s)	β (°)	α (°)	u_∞_ (m/s)	u_res_ (m/s)	β (°)	α (°)	u_∞_ (m/s)	u_res_ (m/s)	β (°)	α (°)
3	3.057	11.06	56.44	3	3.221	21.35	54.65	3	3.478	30.39	46.61
4	4.056	9.51	57.99	4	4.219	18.53	57.47	4	4.477	26.69	50.31
5	5.057	8.58	58.92	5	5.222	16.78	59.22	5	5.488	24.34	52.66
6	6.058	7.95	59.55	6	6.230	15.60	60.40	6	6.505	22.73	54.27
7	7.060	7.50	60.00	7	7.239	14.75	61.25	7	7.526	21.55	55.45
8	8.074	7.75	59.75	8	8.291	15.23	60.77	8	8.641	22.22	54.78
9	9.076	7.43	60.07	9	9.301	14.61	61.39	9	9.663	21.35	55.65
10	10.089	7.64	59.86	10	10.353	15.01	60.99	10	10.778	21.91	55.09

**Table 4:** The CFD velocity vector with turbine rotation in 3D fluid field analysis.

Turbine Blade Location and Its CFD Flow Field	Local Velocity Vectors around the CMOS MEMS Flow Sensor on the Blade	
Root of a blade (Sensor 1) 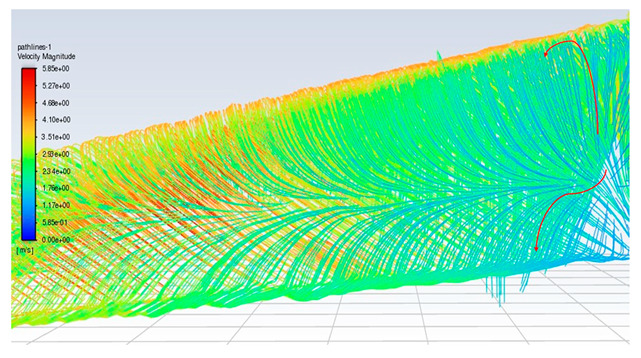	(No spanwise flow)	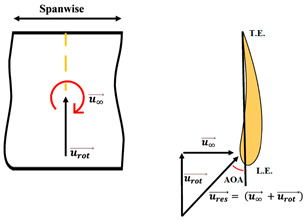
Middle position of a blade (Sensor 2) 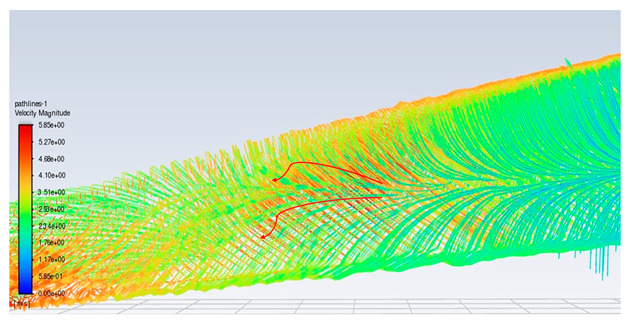	(Upward branch)	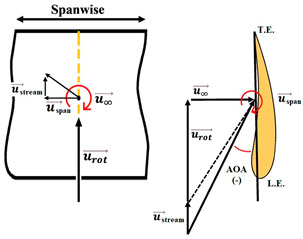
	(Downward branch)	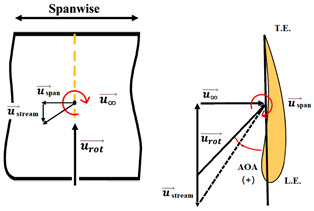
Tip of a blade (Sensor 3) 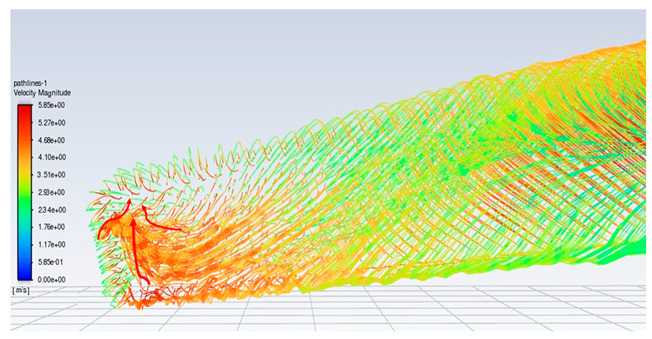	(Tip vortex)	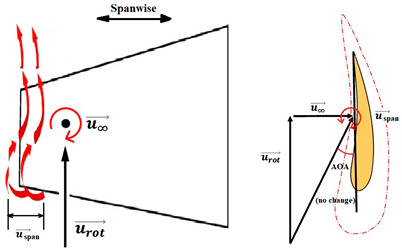

## Data Availability

Dataset available on request from the authors.
